# Nutritional Considerations for Injury Prevention and Recovery in Combat Sports

**DOI:** 10.3390/nu14010053

**Published:** 2021-12-23

**Authors:** Hüseyin Hüsrev Turnagöl, Şükran Nazan Koşar, Yasemin Güzel, Selin Aktitiz, Muhammed Mustafa Atakan

**Affiliations:** Division of Nutrition and Metabolism in Exercise, Faculty of Sport Sciences, Hacettepe University, Ankara 06800, Turkey; nazank@hacettepe.edu.tr (Ş.N.K.); yasmin@hacettepe.edu.tr (Y.G.); selinaktitiz@hacettepe.edu.tr (S.A.); muhammed.atakan@hacettepe.edu.tr (M.M.A.)

**Keywords:** combat sports, nutrition, recovery from injury, sports injuries, supplements

## Abstract

Sports participation is not without risk, and most athletes incur at least one injury throughout their careers. Combat sports are popular all around the world, and about one-third of their injuries result in more than 7 days of absence from competition or training. The most frequently injured body regions are the head and neck, followed by the upper and lower limbs, while the most common tissue types injured are superficial tissues and skin, followed by ligaments and joint capsules. Nutrition has significant implications for injury prevention and enhancement of the recovery process due to its effect on the overall physical and psychological well-being of the athlete and improving tissue healing. In particular, amino acid and protein intake, antioxidants, creatine, and omega-3 are given special attention due to their therapeutic roles in preventing muscle loss and anabolic resistance as well as promoting injury healing. The purpose of this review is to present the roles of various nutritional strategies in reducing the risk of injury and improving the treatment and rehabilitation process in combat sports. In this respect, nutritional considerations for muscle, joint, and bone injuries as well as sports-related concussions are presented. The injury risk associated with rapid weight loss is also discussed. Finally, preoperative nutrition and nutritional considerations for returning to a sport after rehabilitation are addressed.

## 1. Introduction

Combat sports are characterized by repeated movements, including striking (kicking, punching, and blocking defensive moves with the arms and legs), grappling, (wresting an opponent to the ground or using a submission hold), and combinations of these techniques [1]. Popular at competitive and recreational levels all over the world, combat sports improve physical fitness (e.g., increased muscle strength, flexibility, and balance), psychological aspects (e.g., self-esteem, self-awareness, and self-respect), and cognitive function [1].

Sports participation is not without risk, and most athletes incur at least one injury throughout their careers. Olympic combat sport athletes sustain, on average, one injury every 2.1 h of competition [2]. Furthermore, about 30% of the injuries sustained during competition result in >7 days of absence, and about 20% resulted in 1–7 days of absence from competition or training [2]. The treatment and rehabilitation processes have a significant psychological and economic burden on the athlete, family, and society. It is extremely important to implement preventive measures such as progressive training, protective equipment use, regulation of the competition rules, and nutrition in relation to the consequences of injuries. Given that injuries are inevitable despite every precaution being taken, improving the treatment and rehabilitation process and thus decreasing the recovery time remains a fruitful area of research.

Along with its effects on the physical and psychological health of the athlete, nutrition is a pivotal factor affecting sports performance, the level of injury risk, and recovery time from an injury. A significant body of scientific knowledge has been published on nutritional strategies and ergogenic aids to improve sports performance [3,4,5,6]. However, limited research is available about the role of nutrition in reducing the risk of injury and improving the recovery process following injuries in athletes [7,8,9,10,11]. This is because, for athletes who are intent on returning to their sport as quickly as possible, the uncertainties about injury diagnosis and doubts regarding the efficacy of the treatment received are the main concerns of such players. Hence, it is challenging to research such athletes. In recent years, several review papers were published addressing the overall role of nutrition in injury prevention and improving recovery [12,13,14,15]. Some review papers about this issue focused on specific injuries or conditions such as muscle injuries [16,17,18,19], muscle damage [20], and muscle disuse atrophy [21,22], while others focused on various sports branches such as track and field [19,23], football [24], and soccer [19]. Since combat sports are popular all around the world and about one-third of the injuries result in more than 7 days of absence from competition or training, it is worth presenting the role of nutrition in injury prevention and recovery. Therefore, the purpose of this review is to present various nutritional strategies for reducing the risk of injury and improving the treatment and rehabilitation process in combat sports. We will present the role of macro- and micronutrients, total energy intake, and food supplements in the prevention and recovery time for injuries to skeletal muscle, bone, tendons, and ligaments, as well as sports-related concussions (SRCs), the most common injuries in combat sports. Furthermore, the injury risk associated with weight cutting will be discussed along with how to reduce the potential risks. Finally, nutritional considerations for returning to play after rehabilitation will be presented.

## 2. Injuries in Combat Sports

Numerous examples of research are available on the injury prevalence in various combat sports such as wrestling [25,26,27,28], judo [29,30,31], taekwondo [32,33,34,35,36,37], boxing [38,39,40,41,42], and karate [43,44,45], as well as comparisons of injury prevalence among Olympic combat sports [2,46,47,48] and martial arts [1,49]. Due to the heterogeneity of these studies in terms of participants (e.g., elite vs. amateur, children or adolescents vs. adults, and male vs. female), injury definitions, surveillance methods (e.g., self-reporting, medical records, and injury surveillance systems), and characteristics of the reported injuries (e.g., competition injuries vs. training injuries and acute vs. chronic injuries), it is difficult to get an overall view of the injury rate, type, location, and severity. The majority of the published studies present data from the adult population and injuries that occurred during competition. Fortunately, comprehensive findings were reported in a recent prospective cohort study by Lystad et al. [2] that documented the incidence, severity, and profile of injury and the risk factors for injury for Olympic combat sport athletes based on the data from the IOC injury surveillance system and exposure data from official tournament records at three consecutive Olympic Games: Beijing 2008, London 2012, and Rio de Janeiro 2016. In the following sections, we outline a summary of the literature about the injury rate, injury severity, locations, and tissues most frequently injured and the risk factors for injury in Olympic combat sports.

### 2.1. Injury Rate

Olympic combat sport athletes (e.g., wrestling, judo, taekwondo, and boxing) sustain, on average, one injury every 2.1 h of competition [2]. The injury rate per 1000 min of exposure (IIR_ME_) is significantly lower in wrestling (4.8) compared with taekwondo (7.7), boxing (9.2), and judo (9.6), with an overall injury rate of 7.8 [2]. A systematic review and meta-analysis reported that karate athletes sustain, on average, 1 injury every 25 min of competition and most of these injuries are minor or mild in severity [45]. Other reported IIR_ME_ estimates in the literature for Olympic combat sports are 39.2 in karate [45], ranging from 13.8 to 20.4 in boxing [40,50,51], 16.3 in taekwondo [34], 10.9 in judo [52], and 5.9 in wrestling [53]. Jeong et al. [32] reported a 6.9 IIR_ME_ for new or recurrent injuries in junior athletes during the 2018 World Taekwondo Junior Championships. Taekwondo was listed within the top five most injurious sports in each of the Olympic games from 2008 to 2016 [54,55,56].

### 2.2. Injury Severity

According to the study by Lystad et al. [2], about 50% of injuries sustained during competition result in at least 1 day of absence from training or competition. The proportion of injuries leading to >7 days of absence from competition or training is higher in wrestling (39.6%), judo (35.9%), and taekwondo (32.5%) than in boxing (21.0%) [2]. However, the greatest injury burden risk was found in judo when both the incidence and severity were taken into consideration [2]. The incidence rate for time-loss injuries in top-level karate athletes was reported to be relatively low (10%) based on the data collected at four consecutive World Karate Championships (2010, 2012, 2014, and 2016), and these injuries were due to fractures (41%), dislocations (20%), or concussions (12%) [44].

### 2.3. Injury Location

Although the distribution of injury with respect to body location has great variability across combat sports, the most frequently injured body regions are the head and neck (35.9%), upper limbs (31.1%), and lower limbs (26.3%) [2]. Head and neck injuries are most common in boxing (62.1%), upper limb injuries are frequently observed in judo (42.6%), and lower limb injuries are frequent in taekwondo (59.5%) and wrestling (45.5%) [2]. Another study by Lystad et al. [45] reported that the head and neck were the most commonly injured body regions in Olympic-style karate (57.9%), while contusions (68.3%) and lacerations (18.6%) were the most frequent injury types. According to a systematic review [46] based on nine studies of Olympic combat sports, the most frequently injured areas were the head and face (45.8%), wrist (12.0%), and lower back (7.8%) in boxing, the fingers (22.8%) and thigh (9.1%) in taekwondo, the knee (24.8%), shoulder (17.8%), and head or face (16.6%) in wrestling, and the lower back (10.9%), shoulder (10.2%), and knee (9.7%) in judo. In junior taekwondo athletes, the most frequently reported injuries were contusions, ligament ruptures and sprains, and lacerations and fractures to the lower extremities, head, and trunk [32].

### 2.4. Injured Tissues

According to the data obtained at three consecutive Olympic Games (Beijing 2008, London 2012, and Rio de Janeiro 2016), overall, the most commonly injured tissue types in combat sports are superficial tissue and skin (42.9%), in addition to ligament and joint capsule (31.4%) injuries [2]. Overall, joint sprains (31.4%) were the most frequent injury type, followed by lacerations and abrasions (23.2%) and contusions (19.7%), while concussions accounted for 3.2% of all recorded injuries [2]. Similarities and differences exist in the distribution of injuries by pathology across combat sports. Contusions were common in all combat sports (e.g., boxing: 18.5%; judo: 16.0%; taekwondo: 28.6%; and wrestling: 21.8%). Lacerations were reported to be common in boxing (41.1%), while joint sprains were predominant in judo (47.9%), taekwondo (33.3%), and wrestling (40.0%) [2]. Muscle injuries were most common in taekwondo (9.5%), followed by wrestling (9.1%), boxing (5.6%), and judo (5.3%) [2]. Ligament or joint capsule injuries were most common in judo (47.9%), followed by wrestling (40%), taekwondo (33.3%), and boxing (14.5%) [2]. Moreover, a high risk of mild traumatic brain injury (mTBI) or SRCs in combat sports athletes, particularly wrestlers, was reported. As such, the mean annual incidence of mTBI presented to emergency departments in the United States due to wrestling was significantly higher (269.3 per 100,000 person-years) than boxing (85.6 per 100,000 person-years) and martial arts (61.0 per 100,000 person-years) [48].

### 2.5. Risk Factors for Injury

Gender, weight categories, training status, nutrition, wearing protective equipment, and competition regulations are potential factors affecting injury risk in combat sports. Injury risk was reported to be similar between genders and among weight categories in Olympic combat sports. However, a higher IIR_ME_ was observed in athletes who lost compared with their winning opponents [2]. Similarly, when adjusted for exposure time, no significant difference in the injury rates was observed between males and females in Olympic-style karate [45]. However, junior male taekwondo athletes incurred more injuries than females, and contact between the athletes was reported to be the most common cause of injury [32].

## 3. Injury Risk Associated with Rapid Weight Loss (RWL)

Combat athletes compete in specific weight categories to match competitors of similar body weight, size, and strength [57,58]. Many combat athletes believe that by achieving rapid weight loss (RWL) a few days before weight categories are determined for the competition, they gain an advantage over weaker competitors in terms of size and strength [33,57,59]. For this purpose, they lose approximately 2–10% of their body weight in the last 2–3 days before the competition [60]. RWL methods include severe limitation of fluid and food intake, exercise with thermal clothing, sauna, diuretics, weight loss pills, low-carb diets, laxatives, and vomiting [59,61,62]. All these methods may lead to several health problems, an increased risk of injury, as well as a decrease in performance [60,61]. The list of health-related issues associated with RWL is long and includes both acute and chronic effects. Interested readers may be referred to a recent review by Artioli et al. [60]. In brief, the most obvious consequences of RWL methods are dehydration and insufficient intake of nutrients, which in turn may lead to a decrease in blood plasma volume, an increase in heart rate, impaired cardiac functions and thermoregulation, electrolyte imbalances, low oxygen consumption, slowing of fluid passage through the kidneys, and a decrease in glycogen stores [63,64]. In addition, RWL might result in hormonal imbalance [65,66]. All of these altered physiological functions may increase the vulnerability of the athletes to injury. Furthermore, RWL methods increase the risk of injury by negatively affecting cognitive functions such as memory and concentration [29,58] and psychological aspects [65,67]. For example, increased confusion, rage, fatigue, depression, and isolation, as well as decreased short-term memory, vigour, concentration and self-esteem, have been reported in athletes undergoing RWL practices [58,65]. All these psychological changes may result in distractions, lack of control, aggressive behavior, difficulty in decision making, and an inability to follow instructions during the match, all of which has the potential to decrease performance and increase injury risk. Indeed, some epidemiological [68], as well as experimental [69] data, show that RWL might increase the risk of injury. For instance, Korean wrestlers had significantly higher injury rates during periods of RWL (23.18%) compared with other training periods (11.93%) [70]. Most of the injuries occurred in the lower extremities (38.0%), followed by upper extremity (25.9%), trunk (24.8%), and head and neck injuries (11.3%). One study [70] reported major ligament-joint and muscle-tendon injuries following RWL methods. In a study conducted with judokas [29], only 14% of the athletes who did not use RWL methods were injured, while 48.7% of the judokas who applied RWL were injured. Furthermore, a higher risk of injury was observed in athletes who lost 5 kg or more of body weight [29]. In a case study, Oöpik et al. [69] reported that a 5–6% reduction in body weight within 3 days affected wrestlers’ metabolisms and quadriceps femoris muscle function, thus increasing the risk of injury. Similarly, Green et al. [29] reported a higher probability of injury during competition in athletes who had lost more than 5% of their body mass. Limited research is available [29,69,70] in the literature that examines the relationship between RWL habits and the risk of injury. Thus, more research is needed on this topic. Athletes should be advised to avoid RWL strategies to reduce the risk of injury. Experts recommend that the use of RWL methods be banned because they meet all three of the World Anti-Doping Agency (WADA)’s criteria for banning a substance or method from sports (potential to improve performance, potential health risk, and violation of the spirit of sport) [60].

## 4. Nutrition in Muscle Injuries

Muscle injuries are one of the most common sports injuries, leading to decreased physical activity and immobilization, depending on the severity. In the following subheadings, we present various nutritional and ergogenic support strategies to prevent muscle loss and promote injury healing. In this respect, due to their therapeutic roles in preventing muscle loss and anabolic resistance as well as promoting injury-healing amino acid and protein intake, antioxidants, creatine, and omega-3 are given special attention.

### 4.1. Protein and Amino Acid Intake

Increasing protein intake during recovery is one of the first strategies in reducing muscle loss and accelerating the healing process during the injury period [14]. In particular, protein intake should be increased to prevent both muscle loss and anabolic resistance to protein during immobilization after injury [22]. In this respect, the main considerations are increasing daily protein intake in combination with exercise, balanced distribution of protein intake during the day, and the amino acid content of protein. Increasing protein intake to 2.3 g/kg/day is known to reduce muscle loss in states of negative energy balance [71]. For this reason, current daily protein intake recommendation (1.4 g/kg/day) of combat athletes [72] can be increased during the injury period. On the other hand, it should be noted that increasing protein intake to 1.6 g/kg/day in women on bed rest for 60 days was shown not to prevent muscle loss, while a positive effect was observed when combined with aerobic and resistance exercises [73]. Therefore, it is important to emphasize that protein intake can prevent muscle loss, especially when combined with exercise interventions during the rehabilitation process.

In addition to total protein intake, balanced distribution of the daily protein intake in each meal is also important. For example, consumption of a moderate amount of protein in each meal (breakfast: 31.5 ± 1.3 g, lunch: 29.9 ± 1.6 g, and dinner: 32.7 ± 1.6 g of protein) stimulated 24-h muscle protein synthesis 25% more effectively than skewing the protein intake toward the evening meal (breakfast: 10.7 ± 0.8 g, lunch:16.0 ± 0.5 g, and dinner: 63.4 ± 3.7 g of protein) [74]. In another study, it was reported that consumption of 20–25 g (0.30 g/kg) provided more muscle protein synthesis compared with consumption of 0, 5, and 10 g and similar to that for 40 g [75]. Thus, consuming such amounts of protein intake (20–25 g/meal) should be recommended for healthy athletes. However, the amount of protein in meals should also be increased for further protein synthesis during the injury period. When increasing protein intake, the amino acid content should also be considered. In particular, the amount of leucine, one of the keystones of protein synthesis, is important [76], given that leucine helps anabolic stimulation by overcoming resistance to protein synthesis. Indeed, leucine intake was reported to delay muscle loss in rats during immobilization [77]. However, the effects of extra leucine intake in immobilized humans are not incompletely explored. Additionally, future research is needed to determine the amount of leucine to be consumed by athletes during the rehabilitation process.

### 4.2. Creatine

In addition to the well-documented effects of creatine, including improving strength, increasing lean muscle mass, and helping the muscles recover more quickly during exercise, creatine can also be beneficial in reducing muscle atrophy in athletes during immobilization after injury [78]. For example, Hespel et al. [7] reported that consumption of creatine monohydrate (from 20 g down to 5 g daily) in combination with rehabilitation exercises for 10 weeks (3 days/week) accelerated muscle hypertrophy in disuse atrophy induced by 2 weeks of leg immobilization [7]. In another study, Op’t Eijnde et al. [9] investigated the effects of creatine monohydrate consumption on muscle glucose transporter type 4 (GLUT4) levels during 2 weeks of immobilization and the following 10 weeks of rehabilitation training with resistance exercises [9]. Creatine monohydrate consumption of 20 g/day during the immobilization process attenuated the decrease in GLUT4 levels, and consumption of creatine monohydrate during the training period (15 g for the first 3 weeks and 5 g for the last 7 weeks) increased the GLUT4 levels by 40% [9]. Taken together, loading and maintenance doses of creatine can be recommended during immobilization and rehabilitation for the maintenance of muscle mass and metabolic health.

### 4.3. Omega-3

Recent research shows that fish oil plays a significant role in preventing muscle loss. For example, the consumption of fish oil pills for 10 consecutive days in immobilized rats alleviated muscle atrophy by stimulating the protein kinase B pathway in the immobilized soleus muscle [79]. Intake of fish oil for 5 weeks also improved insulin sensitivity in muscle protein metabolism in red angus [80]. However, no study to date has investigated whether a similar beneficial effect of fish oil pills is evident in immobilized humans. Of the limited studies, Smith et al. [81] reported increased anabolic responses to insulin and amino acids following 8 weeks of omega-3 intake in healthy young and middle-aged individuals. In physically active individuals, the consumption of 3900 mg fish oil (containing 3 g n-3) for 4 weeks decreased muscle damage [82]. Although fish oil consumption is promising in reducing muscle loss due to muscle injuries, more studies involving human participants are needed.

### 4.4. Antioxidants

Due to therapeutic roles in improving exercise-induced muscle damage, there has been a significant scientific inquiry into antioxidants. In particular, antioxidants such as vitamins C and E come to the fore in the fight against increased production of reactive oxygen species after muscle injury [83]. However, there are contradictions in the literature regarding the intake of antioxidants after muscle damage, as the therapeutic effects of antioxidants in conditions such as inflammation, loss of strength, fatigue, and cell damage have not been fully elucidated yet [84]. Moreover, some research reported increased muscle damage when antioxidants were consumed following muscle damage due to the prevention of cellular adaptation after exercise [85].

Taken together, although there is no study directly examining the effects of nutrition on muscle injuries in combat sports, it is recommended to increase the daily protein and leucine intake, consume creatine at a loading and maintenance dose, and intake omega-3 supplements to reduce muscle loss.

## 5. Nutrition in Joint, Connective Tissue, and Tendon Injuries

Joint, tendon, and connective tissue injuries are among the most frequent injuries after superficial tissues and skin injuries in combat sports [2]. These injuries can impair performance and lead to immobilization. The repair of joints, tendons, and ligaments is not fully elucidated compared with muscle repair [86], yet current evidence suggests that Collagen supplements and supplements that support collagen synthesis, such as gelatin and vitamin C, are the most prominent strategies in these cases.

### 5.1. Collagen

Collagen, a connective tissue protein, is the primary component of tendons and ligaments [87]. Collagen is rich in glycine, proline, hydroxylysine, and hydroxyproline, and consumption of these amino acids increases collagen synthesis [88] as well as improves the ligament–tendon structure [89,90]. Though collagen synthesis in tendons and muscles is not affected by the increase in amino acid intake [91], consumption of an amino acid content rich in proline, hydroxylysine, and hydroxyproline improves collagen synthesis [92]. Indeed, cell culture studies showed that hydrolyzed collagen significantly increases type 2 collagen biosynthesis as well as stimulates the regulation of the collagen cycle in cartilage tissue [93]. In an animal study, it was shown that oral intake of hydrolyzed type 1 collagen peptides improved the size and composition of collagen fibrils in a tendon cell [94].

Collagen also plays a protective role in joint and connective tissue health, as well as reducing joint pain. For example, consuming 25 mL of liquid collagen containing 10 g of hydrolyzed collagen per day for 24 weeks improved joint health and reduced the risk of joint degeneration and joint pain compared with a placebo [8]. In athletes with chronic ankle injuries, significant reductions in the number of ankle injuries were observed following ingestion of 5 g of collagen peptide for 6 months [11]. Collectively, intake of 10–15 g of hydrolyzed collagen per day appears to be an effective strategy for the prevention and treatment of joint, tendon, and ligament injuries.

### 5.2. Gelatin

Ample evidence shows that gelatin stimulates collagen synthesis. Gelatin, obtained by boiling the skin, bones, tendons, and ligaments of animals such as cows, pigs, and fish, has the same amino acid content as collagen [23,86]. Gelatin was reported to be beneficial in collagen production and returning to the field from an injury [86]. For example, the consumption of 15 g of gelatin 1 hour before a 6-min jump rope test performed to stimulate collagen synthesis was shown to increase collagen synthesis twofold by raising the collagen-1 amino terminal pro-peptide in the blood [95]. When the intake of 15 g of hydrolyzed collagen and gelatin was compared in the same protocol, a similar positive effect was found for the procollagen levels [96], suggesting positive and similar effects for the consumption of 15 g of gelatin or hydrolyzed collagen 30–60 min before exercise [95,96].

### 5.3. Vitamin C

Consuming vitamin C together with collagen amino acids also improves collagen synthesis [92,95]. Vitamin C is a pivotal player in collagen synthesis, and its deficiency causes scurvy, which in turn results in collagen loss. Vitamin C is a cofactor of proline 4 hydroxylase, an enzyme involved in proline hydroxylation and procollagen synthesis in connective tissues [97]. Furthermore, adequate vitamin C intake with collagen is of significant importance given that vitamin C provides an increase in collagen synthesis, tendon and ligament repair, and improvement in surgeries. At least 46 mg/day of vitamin C intake is the minimal dose required to maintain collagen production [23], but the effects of a higher intake on collagen synthesis remain to be determined. Citrus fruits, strawberries, kiwi, peppers, and broccoli are some of the foods rich in vitamin C that should be consumed [98]. Of the limited studies available, Shaw et al. [10] investigated the effects of 24 weeks of evidence-based supplementation of gelatin (10 g/day) and vitamin C (250 mg/day) along with creatine (loading: 20 g × 5 days immediately postoperative, then 3 g/day) and leucine (3 g/day) on the leg strength and muscle mass in two elite rugby union players with ruptured their left anterior cruciate ligaments, which were repaired with traditional hamstring grafts. They reported that rehabilitation programs combined with this type of nutrition strategy could attenuate leg strength loss due to 24 weeks of physical inactivity. Among the very limited studies investigating the therapeutic role of nutrition in joint, connective tissue, and tendon injuries, none were conducted with combat athletes. However, the consumption of 10–15 g of collagen or gelatin containing vitamin C following a joint-connective tissue injury is likely to provide significant support for the prevention and treatment of these injuries.

## 6. Nutritional Strategies in Bone Injuries

Since combat sports involve striking, throwing, or immobilizing an opponent, the main cause of injuries in combat athletes is mechanical energy leading to musculoskeletal injuries [2,99]. Studies reported that the proportion of fractures is greater in mixed martial arts (27%), wrestling (21.3%), and kickboxing, in which fracture rates may reach 20% [100,101]. In addition, one of the most common bone injuries in athletes is stress fractures, which are different from contact fractures [102]. Stress fractures are overuse injuries of the bone that result from the repeated application of stress lower than that required to fracture the bone in a single loading session [103]. Although the pathophysiology of stress fractures is not fully understood, it is considered to occur due in part to insufficient nutrient intake [104].

### 6.1. Vitamin D and Calcium

Although there is a limited number of studies that investigated the association of diet with prevention of or recovery from bone injuries, it is fairly well-known that diet exerts a profound effect on bone health. For example, providing sufficient calcium and vitamin D plays a significant role in optimal bone formation during healing from fractures. Additionally, Barker et al. [105] reported an association of low vitamin D levels with impaired recovery from knee surgery. It was demonstrated that if an individual has sufficient vitamin D, supplementation may be detrimental, especially when used after a bone fracture because certain macrophages might be suppressed [106]. Lappe et al. [107] documented that supplementation with 800 IU/day of vitamin D and 2000 mg of calcium reduced the risk of developing stress fractures in female navy recruits. However, an excessive amount of micronutrient intake during recovery from injury remains a matter of debate. Protein, magnesium, phosphorus, potassium, and fluoride play important roles in bone health [108]. Iron, zinc, silicon, vitamin A, vitamin K, and vitamins C and B are other nutrients supporting metabolic processes important for bone tissue [109,110]. From this point of view, adequate intake of dairy products, fruits, and vegetables, specifically green leafy vegetables, is beneficial a source for the main nutrients that support bone health.

### 6.2. Energy Intake

One of the more specific considerations in nutritional strategies for preventing bone injuries in combat sports is RWL practices [58]. As discussed above, RWL practices may impair athletes’ health in multiple ways. A common practice of RWL in weight-classed athletes is to extremely reduce dietary intake, which might result in a hormonal imbalance [65,66], which in turn acutely affects bone metabolism. Hence, there are concerns that dietary strategies used by athletes to achieve RWL are very likely to lead to a low energy availability (EA) state and nutrient deficiency, which are known to negatively affect bone metabolism and increase the risk of bone injuries [111,112,113]. Low EA is a state of insufficient energy intake to support the physiological functions required to maintain optimal health [114], and it can be observed in both female and male athletes [112]. Ihle and Loucks [115] reported that an energy intake under a threshold of 30 kcal/kg of fat-free mass (FFM) daily results in disruptions in the hormonal markers of the reproductive system and bone metabolism in females. Given that combat athletes lose weight several times a year, their bone health will be negatively affected. Therefore, maintaining an EA of 45 kcal/kg/FFM per day is recommended in order to optimize bone health and prevent bone injuries.

There has been an increase in the popularity of low-carbohydrate diets among athletes in recent years [116]. This remains a matter of debate, as carbohydrate intake provides the largest contribution to the daily total energy intake in the athlete’s diet. Nevertheless, losing weight by manipulation of glycogen stores is a common method used among combat athletes [61]. Furthermore, it is worth noting that these dietary approaches are likely to increase the risk of a low-EA state due to carbohydrate restriction. Although some recent data have shown that low carbohydrate availability might negatively affect bone health independent of EA [117], more research is needed on this topic.

### 6.3. Protein Intake

Protein should be also considered when recovering from bone injuries. Athletes are generally recommended to consume protein at a rate of more than 0.8 g/kg/body mass daily [118]. Considering that protein is one of the key components in bone, it is important for athletes to consume sufficient amounts of protein. On the other hand, some research suggested that high protein consumption could be detrimental to bone due to the associated acidic load [119]. In contrast, recent meta-analyses that investigated the effect of protein on bone health showed that there were no adverse effects of higher protein intake on bone health [120,121]. Instead, there might be some beneficial effects on bone health, particularly when combined with a sufficient calcium intake [23]. On the other hand, bone collagen synthesis, an important factor for bone healing, responds to increased amino acid levels [122]. Studies reported that protein supplementation enhanced recovery from hip fracture surgery [123,124]. However, since these results are based on data obtained from elderly subjects, further studies should investigate whether these effects of protein supplementation are present in healthy athletes.

## 7. Nutrition in Sports-Related Concussions (SRCs)

SRCs are mild traumatic brain injuries (TBIs) global health concern and caused by a biomechanical force to the body which causes temporary deterioration of neurological functions [125]. TBIs are common in contact sports [126,127]. According to the position statement released by the American Medical Society for Sports Medicine about concussions in sports, protective equipment does not provide sufficient protection from the incidence or severity of concussions in sports [128]. Hence, there is a need for divergent strategies capable of preventing or reducing the deleterious effects of SRCs and subconcussive impacts. From this point of view, nutritional supplementation could be valuable when provided before and after TBIs, and this is a fruitful area of research for future studies.

Nutritional recommendations for TBIs are centered around antioxidants and anti-inflammatory agents [125,129]. When a TBI is sustained, neuroinflammation and dysregulation of sodium (Na^+^), potassium (K^+^), and calcium (Ca^2+^) ions occur, and the influx of Ca^2+^ ions leads to oxidative stress [130]. Curcumin, an anti-inflammatory compound, was shown to be effective in reducing the levels of oxidized proteins and normalizing brain-derived neurotrophic factor (BDNF) in rats following TBIs [131]. In addition, there are many studies investigating the effects of n-3 fatty acids on TBIs [132,133,134]. Data from animal studies demonstrated that supplementation of n-3 fatty acids decreased neuronal damage and inflammation and normalized BDNF and neurotransmitter levels [132,133,134]. Although both n-3 fatty acids and curcumin seem to be effective in the treatment of TBIs, solid recommendations cannot be made, and further human research is needed. A review by Trojian et al. [129] suggested that lower serum levels of vitamins D, C, or E before injury worsen the outcomes in animal studies and that caffeine intake after SRCs could be detrimental. For a detailed discussion on nutritional supplements for the treatment and prevention of SRCs, readers may be referred to the recent reviews by Trojian et al. [129] and Lust et al. [125].

## 8. Preoperative Nutritional Strategies

Both the inflammatory response to the injury and surgical stress lead to catabolic reactions [135]. The release of injury-induced stress hormones and proinflammatory cytokines may sustain a prolonged catabolic state characterized by nitrogen loss [136] and insulin resistance [135]. Hyperglycemia occurs despite elevated levels of insulin, exacerbating the depletion of muscle protein [135]. Thus, adequate intake of macro- and micronutrients is required to support this hypermetabolic state and the injury-healing process [12]. Traditionally, preoperative nutrition was mainly focused on recommendations about fasting and restricted fluids beginning at midnight of the day of the operation [137]. An empty stomach minimizes the risk of pulmonary aspiration. However, more recent data showed that these fasting routines can be replaced with less strict ones without putting patients at risk [12,135]. Furthermore, providing high-carbohydrate beverages to patients immediately before operations was shown to be safe and to reduce the catabolic stress of surgery [138,139]. Indeed, nutritional supplements known to support the immune system, which in turn diminishes the catabolic response to operation and maximizes recovery potential, are safe for use before surgery [12,138]. On the operation day, a liquid consisting of high molecular weight starch and essential amino acids is recommended 2 or 4 hours prior to surgery [12]. In addition, given that midnight fasting results in significant depletion of the glycogen stores known to increase the demand for amino acids, preoperative carbohydrate drinks may reduce the risk of insulin resistance by maintaining and improving the whole-body protein balance and muscle function [138].

Moreover, hyperglycemia may increase the risk of postoperative complications. In this regard, a 100-g glucose intake with an oral solution the night before surgery and 50 g of glucose 2 h before surgery were reported to reduce postoperative hyperglycemia [12,139]. In addition, modified waxy maize starch could be used as a preoperation supplement to help maintain glucose levels during surgery as well as to prevent postoperative hypoglycemia. In summation, it is important for athletes to consume adequate complex carbohydrate sources and proteins the night before surgery to minimize postoperative complications.

## 9. The Role of Nutrition in Returning to a Sport Following Injury

Injuries are prevalent in combat sports, and many injuries lead to time loss and absence from sports, including both training and competing. The return of athletes to sports following injury will depend on the injury classification and the cooperation of different disciplines, including sports medicine, sport psychology, and sports nutrition. Although nutritional interventions are often overlooked and not commonly a standard of care in rehabilitation interventions, providing athletes with appropriate sports nutrition consulting during the injury process is a pivotal factor that can augment the recovery process and support healing. Hence, nutritional interventions should be coordinated with the different phases of the recovery process to optimize the healing process. In this section, we will focus on nutritional strategies that play important roles in the rehabilitation phase for athletes following an injury.

### 9.1. Nutrition in Immobilization and Atrophy

Periods of immobilization caused by injuries are often associated with the loss of muscle mass and strength, which are deleterious for health and performance [140,141]. Despite studies reporting muscle disuse atrophy following long experimental periods ranging from 2 to as long as 17 weeks of bed rest or limb immobilization [142,143], some studies reported profound impacts from only a few days of disuse on the skeletal muscle mass and strength [141,144]. Therefore, muscle mass recovery following injury-induced atrophy could be critical for combat athletes to accelerate a safe return to sports. Although one of the best ways to recover muscle mass is exercise interventions (i.e., resistance training), this is not always applicable. Therefore, reliable nutrition strategies can help to limit muscle wasting and maintain muscle mass in these situations [145]. Indeed, adequate total energy intake and appropriate dietary intake are the main nutritional strategies during the rehabilitation of injured athletes, along with the intake of some supplements that have the potential to help [23].

It is well-documented that muscle protein breakdown accelerates during injury recovery to support increased protein requirements due to wound healing and tissue rebuilding [12,146]. However, optimizing healing is more dependent on the intake of protein than the bodily breakdown of protein. Therefore, increasing dietary protein seems to be an effective strategy during this process. Furthermore, given that the decrease in lean body mass is of particular concern due to its role in all protein synthesis necessary for healing, nutritional strategies, especially increasing protein intake, have key importance both to increase lean body mass synthesis and to improve healing by increasing protein synthesis [146]. However, research yielded equivocal results, with some studies reporting benefits [147,148] while others reported no benefits [149,150]. For example, a recent systematic review by Pasiakos et al. [151] reported that protein supplementation did not enhance the recovery of muscle function during recovery, suggesting that sufficient dietary protein provided in the general diet of an athlete might be enough and with no additional protein intake required to prevent muscle injury. It is noteworthy that despite the findings of this study, increased dietary protein is an adjunct therapy to attenuate muscle atrophy and promote repair following an injury, which in turn accelerates a safe return to sports [23]. Collectively, injured athletes should consider maintaining protein intake to reduce muscle mass loss during the healing process [152].

There are several other nutrients known to promote healing following an injury, including vitamins C, D, and E, creatine, glycine, polyphenols, flavonoid, and branched-chain amino acids (BCAAs). Although it is not fully explored, some researchers advocate that vitamins C, D, and E work either by acting as an antioxidant or through a reduction in inflammation [23]. For example, it was shown that vitamins C and E can decrease recovery time, but it remains to be elucidated whether these vitamins can optimize the healing process [153,154]. Research has also questioned whether increasing dietary glycine could accelerate the healing of tendons in rats [155,156] and marathon runners [157]. The findings showed that a diet containing 5% glycine, which is extremely high and not realistic for consumption, increased collagen and mechanical strength in rats [155]. In contrast, Buncman et al. [157] reported that 10 g of glycine supplementation (3 times/week for 2 weeks) did not heal skeletal muscle injuries in young male and female marathon runners. The use of glycine in athletes following true injury has not been fully explored, and therefore, clinical studies are needed for further insights. Moreover, although there was no consensus reached [158], some studies reported that BCAAs such as leucine, isoleucine, and valine can boost healing after a musculoskeletal injury as well as increase protein synthesis and inhibit protein breakdown [159,160]. In particular, considering leucine is an essential amino acid found in greater amounts in proteins and is required for optimal stimulation of the rate of muscle protein synthesis [161], the ingestion of leucine in a mixture of essential amino acids during healing can reverse an attenuated response of muscle protein synthesis. However, given that there is little research in this area of sports nutrition, clinical studies are needed to determine whether these supplement-based strategies can influence the recovery process of injured athletes. 

### 9.2. An Obvious Nutrient to Be Avoided after Injury: Alcohol

Large amounts of alcohol consumption after training and competition are part of the social aspects of many sporting events [162]. Research has documented that it is very likely that athletes consume alcohol above the threshold classified as hazardous drinking [163,164]. Given that consumption of alcohol is associated with mental distress-related factors [165], one can assume that sports competition and overwhelming training periods might be a factor in the increasing consumption of alcohol among athletes through the effect of these factors. Although many of the molecular processes disrupted by alcohol are typically known, studies investigating the mechanisms of alcohol-induced tissue injury are rare. A complete consideration of the literature is beyond the scope of this review. Thus, interested readers are referred to other reviews [166,167]. Overall, as pointed out by a review paper [13], alcohol is an obvious nutrient best avoided after injury. Accumulating evidence shows that alcohol ingestion can impair muscle protein synthesis and wound healing likely by reducing the inflammatory response [168,169,170] and can increase lean body mass loss during immobilization [171]. It was also shown that alcohol ingestion suppressed the elevated rates of muscle protein synthesis induced by exercise and protein ingestion in young, active males [168] and the fracture repair process in rats [172]. Indeed, alcohol consumption after exercise and injury is likely to have a detrimental effect on the athlete’s recovery due to not eating or resting adequately as a result of intoxication [168,173]. Moreover, it is worth noting that increased blood flow to the injury site is likely to worsen the severity of the injury and adversely affect the rate and outcome of healing. Therefore, abstinence from alcohol consumption to limit blood flow to the site of the injury is routinely advised due to the known vasodilatory effect of alcohol [167,173,174]. Taken together, limited alcohol ingestion among athletes during the training period and healing process is vital for a safe return to sports. The effects of alcohol on recovery and the time to return to sports following injuries in athletes remains a fruitful area of investigation.

### 9.3. Energy Intake Following Injury

The main goal during a phase of restricted training due to injury should be to maintain physical capacity via sustaining cardiovascular capacity and muscle mass and minimization of undesired body fat increases [175]. Following an injury, there is an inevitable reduction in athletic activities, even if not stopped completely, to allow the injuries to recover, which in turn results in reduced energy expenditure (EE). This reduction in EE consequently requires a concomitant reduction in energy intake to prevent gains in body fat [23]. Considering carbohydrates are an essential part of many athletes’ daily energy intake during hard training periods, it would be appropriate to reduce carbohydrate intake during inactivity [176]. However, it should be noted that there should not be a drastic reduction in energy intake for injured athletes, since the healing process leads to a substantial increase in EE [177], especially early and if the injury is severe (i.e., by up to as much as 50%) [13,178], depending on the type and severity of the injury. In particular, the EE of athletes using crutches was reported to increase two- to threefold [179]. Furthermore, given that performing some form of exercise for the non-injured limb(s) is a common practice among athletes, a proper balance between EE and energy intake during the healing period should be sought. Importantly, energy intake during immobilization is known to exert a profound effect on muscle protein synthesis [13]; that is, a negative energy balance would result in reduced muscle protein synthesis and greater losses in functional strength and prolong the time before returning to sports [12]. Therefore, maintaining an energy balance, specifically a high-protein intake, is critical to attenuate the loss of lean body mass. Injured athletes can minimize weight and fat gain while still meeting the nutritional needs of recovery with a diet including a greater proportion of protein in combination with complex carbohydrates [12]. Although weight and fat gain are the main concerns among athletes during the healing process, it would be an appropriate strategy to maintain the macronutrient composition via a diet with a 2:1 carbohydrate-to-protein ratio (e.g., 240 g of carbs and 120 g of protein), which was shown to promote positive changes in body composition [180,181,182]. In this regard, careful planning is needed to ensure that sufficient energy from macronutrient nutrition is consumed during recovery from an injury.

## 10. Conclusions

Injuries will happen to athletes as an inevitable consequence of sports. In this regard, nutrition solutions that can reduce the risk of injury, as well as decrease recovery time, should be carefully implemented. Athletes should be encouraged to seek advice from experienced staff with up-to-date knowledge of nutritional strategies in the athletic population. As a divergent field among sports disciplines, combat sports are contact sports involving one-on-one combat that engage in full contact to score points, to cause an opponent to submit, or to disable an opponent in a contest or match. Because of this nature, the risk of injury is higher in combat sports (i.e., 7.8 injury/1000 min of exposure) compared with most other sports. Therefore, providing evidence-based nutritional strategies that could reduce the risk of injury as well as optimize the healing process is a pivotal factor for minimal risk of injury and a safe return to sports. Furthermore, given that chronic low energy availability is a major risk factor for bone injuries, athletes should maintain their energy balance such that the energy intake (total kcal consumed) equals the EE (total kcal expended). Thus, change in energy demands should be carefully monitored during the training, competition, and recovery periods. Additionally, preventing RWL and consuming sufficient protein are of significant importance for bone health, particularly in anabolic resistance in muscle during combat sports. In addition to the protective effects of anabolic resistance, a sufficient protein intake plays a primary role in the maintenance of lean muscle mass and is an important predictor of muscle strength, power, balance, exercise tolerance, weakness, and fatigue. In this respect, increasing the daily protein intake in combination with exercise, balanced distribution of protein intake among meals, and consuming protein with a high content of leucine will be effective strategies for optimal performance and recovery.

In terms of the maintenance of muscle mass and metabolic health, creatine supplementation seems to improve strength and maintain lean muscle mass during the immobilization and rehabilitation process. Moreover, although the published literature is not as abundant for other supplements as for creatine, there is growing interest in the role of fish oil consumption, calcium, and vitamin C, D, and E supplementation in reducing muscle loss and inflammation associated with injuries during the recovery period. In addition, vitamin C provides an increase in collagen synthesis, tendon and ligament repair, and improvement in surgery outcomes. Thus, an adequate vitamin C intake (≥46 mg/day) should be reached to maintain collagen production. Also, consuming hydrolyzed collagen (10–15 g/day) is an effective strategy for the prevention and treatment of joint, tendon, and ligament injuries. In particular, consumption of gelatin or hydrolyzed collagen 30–60 min before exercise is beneficial for collagen production. Moreover, due to their role in reducing oxidized proteins, neuronal damage, and inflammation as well as normalizing BDNF and neurotransmitter levels in animal models, n-3 fatty acids and curcumin are likely to be effective nutritional supplements during the healing process. Despite accumulating evidence, however, more studies involving humans, specifically athlete populations, are needed to better understand the possible benefits of these supplements for the prevention of injury or recovery of injured athletes.

Notably, as a part of preoperative nutrition, it is important to provide athletes with an adequate amount of macro- and micronutrients and nutritional supplements that support the immune system. This will likely meet the demands of the catabolic state and contribute to the injury-healing process. In this respect, consumption of complex carbohydrates and proteins the night before surgery is recommended to minimize postoperative complications. Additionally, alcohol ingestion should be limited due to its potential adverse effects on muscle protein synthesis and wound healing, as well as weight gain and lean body mass loss during immobilization. Considering the majority of the findings about the role of nutrition in injury recovery come from animal studies or clinical human studies, further high-quality studies, particularly from more diverse populations, specifically athletes, are needed.

## Data Availability

Not applicable.
